# Iodine Accumulation and Tolerance in Sweet Basil (*Ocimum basilicum* L.) With Green or Purple Leaves Grown in Floating System Technique

**DOI:** 10.3389/fpls.2019.01494

**Published:** 2019-12-18

**Authors:** Luca Incrocci, Giulia Carmassi, Rita Maggini, Caterina Poli, Djamshed Saidov, Chiara Tamburini, Claudia Kiferle, Pierdomenico Perata, Alberto Pardossi

**Affiliations:** ^1^Department of Agriculture, Food and Environment, University of Pisa, Pisa, Italy; ^2^Samarkand Agricultural Institute, Samarkand, Uzbekistan; ^3^Plantlab, Institute of Life Sciences, Sant’Anna School of Advanced Studies, Pisa, Italy

**Keywords:** anthocyanic variant, iodine toxicity, leaf antioxidant capacity, plant mineral nutrition, leaf gas exchanges, hydroponic system

## Abstract

Iodine deficiency is a serious world-wide public health problem, as it is responsible for mental retardation and other diseases. The use of iodine-biofortified vegetables represents a strategic alternative to iodine enriched salt for people with a low sodium diet. However, at high concentrations iodine can be toxic to plants. Therefore, research on plant iodine toxicity is fundamental for the development of appropriate biofortification protocols. In this work, we compared two cultivars of sweet basil (*Ocimum basilicum* L.) with different iodine tolerance: “Tigullio,” less tolerant, with green leaves, and “Red Rubin,” more tolerant and with purple leaves. Four greenhouse hydroponic experiments were conducted in spring and in summer with different concentrations of iodine in the nutrient solution (0.1, 10, 50, 100, and 200 μM), supplied as potassium iodide (KI) or potassium iodate (KIO_3_). Plant growth was not affected either by 10 μM KI or by 100 μM KIO_3_, while KI concentrations higher than 50 μM significantly reduced leaf area, total plant dry matter and plant height. The severity of symptoms increased with time depending on the cultivar and the form of iodine applied. Growth inhibition by toxic iodine concentrations was more severe in “Tigullio” than in “Red Rubin,” and KI was much more phytotoxic than KIO_3_. Leaf iodine concentration increased with the iodine concentration in the nutrient solution in both varieties, while the total antioxidant power was generally higher in the purple variety. In both basil cultivars, a strong negative correlation was found between the photosynthesis and the leaf iodine content, with significant differences between the regression lines for “Tigullio” and “Red Rubin.” In conclusion, the greater tolerance to iodine of the “Red Rubin” variety was associated with the ability to withstand higher concentrations of iodine in leaf tissues, rather than to a reduced accumulation of this element in the leaves. The high phenolic content of “Red Rubin” could contribute to the iodine tolerance of this purple cultivar.

## Introduction

Iodine deficiency is a serious world-wide public health problem, as it is responsible for important diseases such as cretinism and goiter. With the exception of sea-derived food, most foods have generally a low iodine content ranging from 30 to 100 µg I kg^−1^ fresh weight, mainly depending on the site of production (Zimmermann, 2017). The adequate Recommended Daily Allowance (RDA) for iodine is 90–120, 150, and 290 µg per day respectively for children, adults, and pregnant or breastfeeding women (EFTSA NDA Panel, 2014).

Since the 1920s, the main strategy to contrast low iodine intake has been the systematic iodination of table salt, which has considerably reduced the incidence of iodine deficiency disorders (Zimmermann, 2017). Anyway, currently 30–38% of the world’s population remains with insufficient iodine intake and is at risk for iodine deficiency (White and Broadley, 2009). Salt iodination alone is insufficient to satisfy the total requirement of iodine for human health, because iodine could be lost from table salt by volatilization (Mottiar and Altosaar, 2011). Moreover, a large part of the population at risk of cardiovascular diseases cannot use iodized table salt (Kiferle et al, 2013). The World Health Organization (2017) has promoted the assumption of iodine through the consumption of seafood and biofortified food such as vegetables, since the iodine contained in these foods can be easily assimilated by humans up to 99% of its amount (Weng et al, 2008).

Although iodine is a not-essential element for plants, it is currently considered beneficial. Plant can uptake iodine by the root or by the stem or leaves through the stomata and/or the cuticular waxes with high degree of unsaturation that are susceptible to iodine addition (Tschiersch et al., 2009). Iodine could reach the aerial plant organs either in dissolved form (like sea aerosol or spray) or as a gas (I_2_ and/or CH_3_I).

Many studies showed that the plant response to iodine application is variable according to the chemical form used (iodide, I^−^; iodate, IO_3_^−^; iodoacetate, ICH_2_COO^−^), the concentration applied, and the growing system (Medrano-Macías et al., 2016; Duborská et al., 2018). Iodine toxicity is reported in the order I_2_(aq) > I^−^> IO_3_^−^ (Mackowiak and Grossl, 1999; Dai et al., 2009). The assessment of iodine toxicity becomes more complicated in the case of cultivation in soil, due to the different stability and interaction of each chemical species with biotic and abiotic soil components.

Unfortunately, relevant information on the uptake mechanism and phytotoxic effects of I^−^ and IO_3_^−^ in higher plants is sparse. In contrast, significant research has been devoted to iodine biofortification, as confirmed by the numerous studies on the application of I^−^ and/or IO_3_^−^ to various crop species. According to a review by Medrano-Macías et al. (2016), most of the work has been conducted on vegetable crops (more than 12 different species), such as lettuce (Smoleń et al., 2014a), spinach (Zhu et al., 2003; Smoleń and Sady, 2012), tomato (Caffagni et al., 2011; Kiferle et al., 2013; Smoleń et al., 2015b), and on cereals such as rice (Kato et al., 2013), wheat (Mao et al., 2014), and barley (Duborská et al., 2018).

To the best of our knowledge, no study on either iodine toxicity or biofortication of sweet basil has been published. Sweet basil (*Ocimum basilicum* L.) is widely cultivated and mostly used for food preparation, especially in Italy and Mediterranean regions (Makri and Kintzios, 2008), and in recent years also purple-leafed variants of sweet basil have been increasingly used, especially for ornamental purposes (Makri and Kintzios, 2008; Pardossi et al, 2015). In particular, the cultivar “Red Rubin” has been extensively characterized for its higher tolerance to boron toxicity compared with green-leafed cultivars (Landi et al., 2013; Landi et al., 2014; Pardossi et al., 2015). Boron toxicity induces leaf chlorosis and necrosis, which reduce photosynthesis and dry matter accumulation (Landi et al., 2013), and both these symptoms are quite similar to those produced by iodine toxicity.

Starting from the above evidences, the aim of the present work was to assess the tolerance to iodine toxicity by two cultivars of sweet basil grown in hydroponic system: “Red Rubin” a purple-leafed variety with high concentration of anthocyanins in the leaf epidermis and the typical green leafed “Tigullio.”

Further aims of this work were to model the relationship between the concentration of I^−^ or IO_3_^−^ in the nutrient solution or in the leaves and the growth inhibition of sweet basil, and to investigate whether the tolerance to iodine toxicity was associated with: i) a reduced iodine uptake and accumulation; ii) an interaction between iodine concentration and the uptake of other nutrients; iii) a reduction of the photosynthetic activity.

## Materials and Methods

### Plant Material and Growing Conditions

Four different experiments were conducted in a glasshouse in Pisa (Tuscany, central-western Italy), with seedlings of two cultivars of sweet basil, “Tigullio” and “Red Rubin.” Seeds were purchased from Franchi Sementi (Milano, Italy). The seedlings were grown in hydroponic culture (floating system) in late spring or in early summer (see **Table 1** for details). Inside the greenhouse, plants were grown under natural light.

**Table 1:** Basic information of the greenhouse experiments conducted in 2015 and 2016 on of two cultivars (“Tigullio” and “Red Rubin”) of sweet basil (*Ocimum basilicum* L.) grown hydroponically with different concentrations and form of iodine in the nutrient solution.

Parameter	Experiment 1	Experiment 2	Experiment 3	Experiment 4
Growing season	June 2015	July 2015	May 2016	May 2016
Hydroponic system	50-L tank	50-L tank	50-L tank	3-L pot
Plants per tank or pot	16	16	16	4
Plant age at transplanting (days from sowing)	18	14	14	14
Plant age at the onset of iodine treatment (days from sowing)	24	21	21	21
Treatment duration (days)	21	16	14	12
Iodine concentrations under investigation				
KI (μM)	10, 100	10, 50,100, 200	50, 100, 200	100, 200
KIO_3_ (µM)	10, 100	–	100, 200, 400	100, 200
Average air temperature (C°)	29.4	32.4	24.1	24.3
Daily average global radiation (MJ m^−2^ day^−1^)	12.6	13.3	10.1	10.4
Determination				
Plant growth	**√**	**√**	**√**	**√**
Mineral element tissue content	**–**	**–**	**√**	**–**
Iodine toxicity modelling	**–**	**√**	**–**	**–**
Iodine tissue content	**–**	**√**	**√**	**√**
Chlorophylls and carotenoids content	**–**	**√**	**√**	**√**
Water uptake	**–**	**–**	**–**	**√**
CO_2_ assimilation (A)	**–**	**–**	**–**	**√**

The seeds of basil were sown using trays with rockwool plugs (Grodan Plug^®^, Grodan Rockwool B.V., Roermond, The Netherlands). After sowing, the trays were maintained five days in a germination chamber with a constant temperature of 25°C and 75% relative humidity. Afterwards, they were moved to the greenhouse for the correct development of the plantlets, which were transferred in the hydroponic system at the stage of four visible true-leaves. In Experiments 1, 2, 3, each hydroponic system consisted of a polystyrene tray floating in a 50-L plastic tank (each tank hosted 16 plants, 8 “Tigullio” plus 8 “Red Rubin”), while 3-liter plastic pots were used in *Experiment 4*, each one hosting 4 plants of the same variety. Planting density was approximately 96 plants m^−2^ of ground area. In all experiments, the nutrient solutions were prepared using tap water and appropriate amounts of salts of analytical grade (Carlo Erba Reagents, Milano, Italy) to avoid iodine contamination. The tap water contained 4.0 mM HCO_3_-, 4.5 mM Cl, 0.25 mM S–H_2_SO_4_, 2.7 mM Ca, 0.8 mM Mg, 2.0 mM Na and negligible concentrations of other nutritive ions, including iodine (0.02 ± 0.01 µM I). The nutrient solution had a pH and an electrical conductivity (EC) of 5.5 and 2.4 dS m^−1^, respectively, and contained the following concentrations of macroelements and trace elements: 10.0 mM N–NO_3_-; 1.0 mM P–H_3_PO_4_; 3.8 mM S–H_2_SO_4_, 8.5 mM K; 4.5 mM Ca; 2.0 mM Mg; 2.0 mM Na; 4.5 mM Cl; 40.0 µM Fe; 25.0 µM B; 10.0 µM Mn; 10.0 µM Zn; 3.0 µM Cu; 1 µM Mo. The tested iodine concentrations ranged from 0.10 ± 0.03 µM (control) and 200 µM (**Table 1**). In each hydroponic system, the large volume of the nutrient solution limited the variations of pH (5.5–6.0), EC (2.3–2.5 dS m^−1^) and ion concentrations, which were checked in all treatments every 2–3 days and remained within 3–5% the initial values throughout the experiments (data not shown).

### Experimental Design

The treatments were defined by a combination of two factors: the concentration of iodine in the nutrient solution and the cultivar (“Tigullio” and “Red Rubin”). In all experiments, the treatments were arranged in a totally randomized design with three replicates, each consisting of a tank or a pot.

The experimental treatments were differentiated one week after transplanting in the hydroponic system by adding potassium iodide (KI) or potassium iodate (KIO_3_) of high purity (purity > 99%; Duchefa Biochemie B.V., Haarlem, The Netherlands) directly to the nutrient solution.

Four experiments were carried out with both basil genotypes.

In *Experiment 1* we investigated the effect of iodine source (KI or KIO_3_) and concentration (0.1, 10 and 100 µM) on plant growth. The experiment was conducted in summer 2015 and lasted 21 days.

In *Experiment 2* KI was tested at the following concentrations: 0.1, 10, 50, 100, and 200 µM. The experiment was conducted in summer 2015 and lasted 16 days.

In *Experiment 3* we studied the effect of the iodine source (KI or KIO_3_) and its concentration in the nutrient solution on plant growth and leaf nutrient content. The following concentrations were used: 0.1, 50, 100, 200 µM for KI, and 100, 200, 400 µM for KIO_3_. The experiment was conducted in spring 2016 and lasted 14 days.

In *Experiment 4* we assessed the effect of 100 and 200 µM KI or KIO_3_ on gas exchange at the two leaves of the 2^nd^ node. The antioxidant capacity and the contents of chlorophylls, carotenoids and total phenols were also assessed in the same leaves, along with the photosynthetic activity (through leaf gas exchange measurements). The experiment was carried out for 14 days in spring 2016.

### Determinations and Measurements

#### Dry Matter and Mineral Ion Determinations in Plant Tissue

The tissues were dried for three days in a 70°C oven to constant mass. After the determination of total dry matter (DW), they were ground in a laboratory mill to a powder. For mineral content determinations, 200 mg oven dried ground samples were wet digested in a mixture of nitric and perchloric acids (HNO_3_:HClO_4_ 5:2 v/v) at 230°C for 1 h. Atomic absorption spectrometry (Varian Spectra AA240 FS, Australia), was used to quantify K, Ca, Mg, Na, Fe, Mn, Cu, and Zn, while spectrophotometry was employed for P determination using the molybdenum blue method (Olsen and Sommers, 1982). Nitrogen was determined by the micro-Kjeldahl procedure (Kacar, 1972); nitrate content was measured by spectrophotometry using the salicylic–sulphuric acid method (Cataldo et al., 1975) after extraction of 100 mg dry samples with 20 ml water for 1 h. The same methods were used to analyze the nutrient solutions.

#### Iodine Determination in Plant Tissue and Nutrient Solution

The leaf iodine concentration was determined on samples taken from the whole leaf biomass (Experiments 2 and 3) or from leaves of the 2^nd^ node (*Experiment 4*).

The concentration of total iodine was determined by inductively coupled plasma mass spectrometry (ICP-MS) both in the nutrient solutions and in plant tissues (200 mg powdered dry matter), according to the official method for iodine determination in foodstuffs (European Standard BS EN 15111:2007), which consisted in an alkaline extraction (90°C for 3 h) with 25% tetramethylammonium hydroxide (TMAH). The samples were filtered and analysed by ICP-MS using tellurium as internal standard. The analyses were carried out by a private laboratory (Pontlab, Pontedera, Pisa, Italy).

#### Chlorophyll and Carotenoids Determination in Leaves

Five disks (12 mm diameter; approximately 0.5 g fresh weight, FW) were sampled from 2^nd^ node leaves, placed in 10-ml test tubes and soaked with 5 ml methanol. The tubes were sonicated 15 min in ice bath four times and stored overnight at −20°C. After the separation of the supernatant, the extraction of the disks was repeated with 5 ml fresh methanol. The two supernatant aliquots were pooled and, after proper dilution (1:5 or 1:10) with methanol, the absorbance of the extracts was read at 665.2, 652.4, and 470 nm. The concentrations of the pigments (µg ml^−1^) were calculated according to Lichtentahler and Buschmann (2001):

(1) [chlorophyll a]=16.72×A(665.2)−9.16×A(652.4)

(2) [chlorophyll b]=34.09×A(652.4)−15.28×A(665.2)

(3) [carotenoids]={1000×A(470)−1.63×[chlorophyll a]−104.96∗[chlorophyll b]}/221

where A (665.2), A (652.4), and A (470) represent the absorbance at the wavelength (expressed in nm) reported in parentheses. The content of total chlorophylls and carotenoids in the tissues was expressed as µg g^−1^ FW.

#### Total Phenolic Content and Antioxidant Capacity

##### Preparation of Plant Leaf Extracts

The determinations of total phenolic content and antioxidant capacity were performed using the leaves at the 2^nd^ node. The analyses were carried out in quadruplicate in methanolic extracts as follows: 0.15 g of leaf tissues were homogenized in a mortar with 5 ml of 70% methanol (v/v) and extracted overnight at 4°C in the dark, under continuous agitation. After centrifugation (5 min, 10,000 rpm at RT) the clear supernatant was collected and used for the subsequent analyses.

##### Total Phenols

The concentration of total phenols was determined using the Folin-Ciocalteu reagent, as reported by Kang and Saltveit (2002). Briefly, 125 µl of leaf methanolic extracts were diluted in distilled water (1:4), mixed with the Folin-Ciocalteau reagent to a final volume of 750 µl and vortexed. After 5 min, 1.25 ml of a 7% (w/v) sodium carbonate solution and 1 ml distilled water were added to the mix, which was left to stand at room temperature in the dark for 90 min. The spectrophotometric readings were carried out at 765 nm and the results were expressed in terms of gallic acid equivalents, on the basis of a standard calibration curve.

##### Antioxidant Capacity

It was evaluated by using the DPPH and the FRAP assays (Dudonné et al., 2009). For DPPH analyses, different volumes of the extract (from 5 to 50 µl) were diluted in 70% methanol and added to a fixed volume (335 µl) of a freshly prepared DPPH radical solution (0.25 mM 1,1-diphenyl-2-picrylhydrazyl in 70% methanol) to reach the final volume of 1 ml; the mixture was vortexed and left to stand at room temperature for 30 min in the dark. The absorbance of the resulting solution was read at 517 nm. A blank solution (control) was prepared by mixing 70% methanol with DPPH solution, and the scavenging activity was determined by the following equation:

(4) $$\%\;scavenging\;activity=100\times (A_{control}-A_{sample})/A_{control},$$

where A is the absorbance at 517 nm.

The tissue antioxidant capacity was expressed as inhibitory concentration (IC_50_ value), which indicates the sample concentration that is required to scavenge 50% of DPPH radical. The IC_50_ value was calculated by plotting the scavenging activity against the concentrations of the samples.

The FRAP assay was carried out mixing 2.0 ml acetate buffer (0.25 M, pH 3.6), 900 µL freshly prepared FRAP reagent (2 mM ferric chloride and 1 mM 2,4,6-tris(2-pyridyl)-s-triazine in acetate buffer), 100 µL plant methanolic extract. A calibration curve was prepared with ferrous ammonium sulfate standard solutions, containing 0–1,000 µM Fe(II). Absorbance was recorded at 593 nm.

#### Plant Water Uptake Measurement

In *Experiment 4*, four basil plants were cultivated in 3-liter pots, and each pot was weighted every 2–3 days without the plants and their floating tray support. After weight determination, the nutrient solution was completely replaced with fresh nutrient solution.

#### Gaseous Exchange Determinations

A CIRAS-2 portable photosynthesis system (PP Systems, Amesbury, MA, USA) was used for the determination of gas exchanges at 500 µmol m^−2^ s^−1^ of Photosynthetic Active Radiation (PAR) intensity, 450 ppm CO_2_ concentration, 65% relative humidity and 27°C leaf temperature, which was close to the greenhouse temperature during the measurements. In particular, CO_2_ assimilation (A), transpiration, leaf stomatal conductance (Gs) and intercellular CO_2_ concentration (Ci) were determined by real-time measurements, which were performed at the opposite leaves of the 2^nd^ node. Three replicates were analysed for each treatment.

### Modelling and Statistics

#### Modelling Growth Response to Iodine Level

The relationship between the nominal iodine concentration in the root zone versus total plant dry biomass or stem length was determined in *Experiment 2* for both cultivars, using the Maas and Hoffman model for crop response to salinity (Maas and Hoffman, 1977). According to this model, the relative growth (DW) of root, stem, leaf or whole plant or the relative stem height (Y*), expressed as the percentage of maximum growth or stem height (*Ymax*), is plotted as a function of the KI concentration in the nutrient solution (*X*). For *X* below a critical threshold value (*t*) no significant difference is observed in Y* (plateau region); thereafter, by increasing *X*, Y* decreases according to a linear function with slope *s* (linear region):

(5) $$Y^{*}=100-s\cdot (X-t)$$

Data of root, stem, leaf and whole plant DW and plant height measured at the end of *Experiment 2* were computed using the procedure described by Magán et al. (2008) to evaluate *Ymax*, *t* and *s*. For each basil genotype, one-way analysis of variance (ANOVA) was conducted with KI as the source of variation, to select the treatments with the highest statistically similar values of root, stem leaf or whole plant DW or stem length. The corresponding average value (*Ymax*) was used to evaluate Y* at each KI level. To determine the values of *s* and *t*, a series of linear regressions was performed to fit the pool of data in the linear region. The regression line with the highest determination coefficient (R^2^), whose slope provided the value of *s*, allowed to evaluate *t* as the KI concentration at which Y* assumed the value *Ymax*.

#### Statistical Analysis

In all the experiments the data were subjected to two-way ANOVA, with the cultivar and the iodine treatment as the sources of variation. Mean values were separated according to the Duncan’s test, at P < 0.05. The program Statgraphics Plus 5.1 (StatPoint, Inc., Herndon, VA, USA) was used to perform the ANOVA and linear regression. A representative run is reported for each experiment, which was repeated two times with similar results.

## Results

### Iodine Phytotoxicity Symptoms

**Figure 1:**
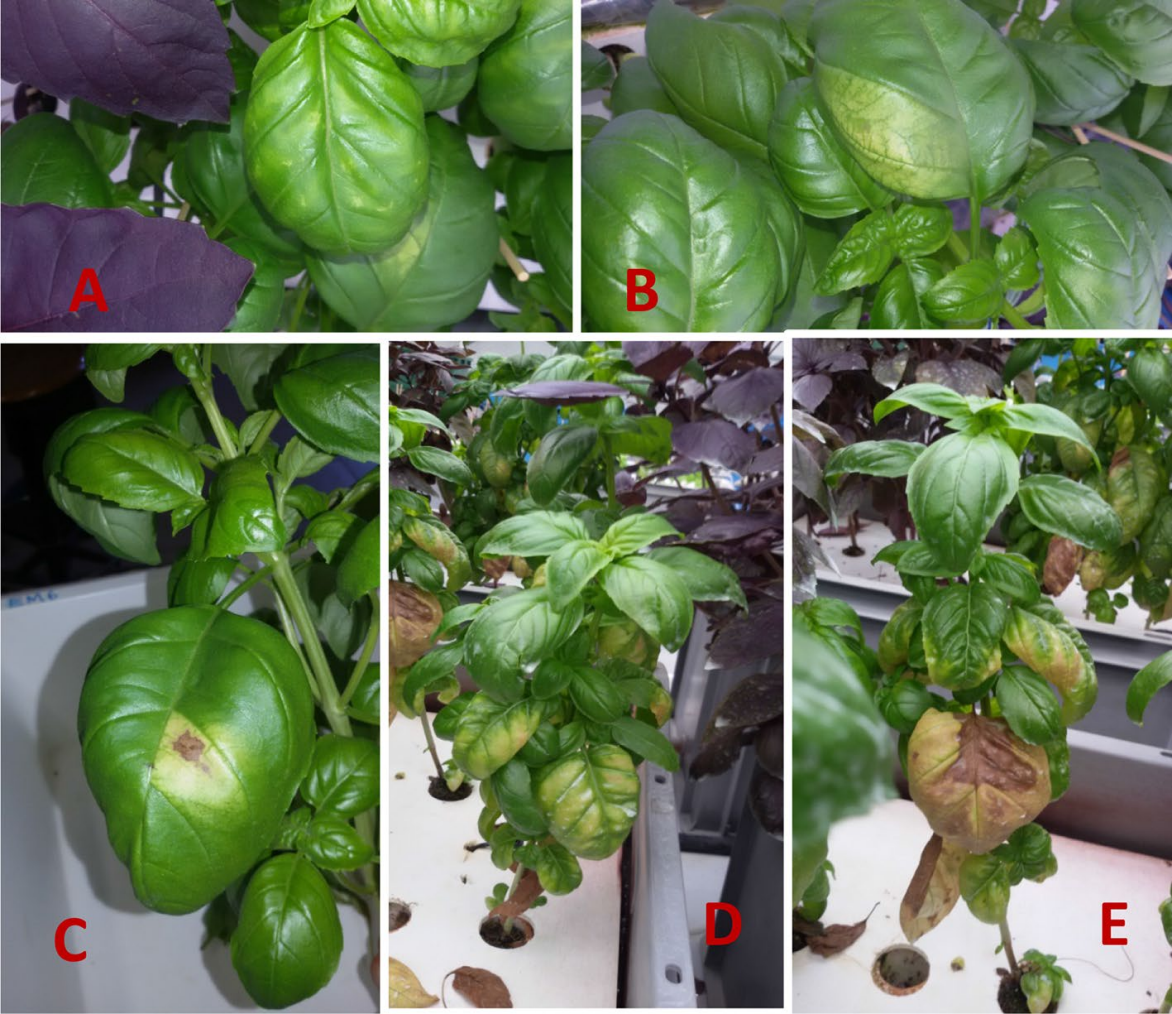
Symptoms of iodine toxicity in sweet basil (*Ocimum basilicum* L., cultivar “Tigullio”) grown hydroponically for 14 days from 9^th^ to 23^rd^ May 2016 (*Experiment 3*) with 200 μM KI in the nutrient solution. **(A** and **B)** general chlorosis and yellow interveinal patches three days after the onset of iodine treatment. **(C)** after one week of iodine treatment brown necrotic spots of basal leaves after seven days. **(D** and **E)**: severe leaf chlorosis and necrosis, and leaf drop after 14 days.

In all the experiments, the symptoms of iodine toxicity appeared within three days of exposure as a reduction of stem elongation, leaf expansion and root development, and within one week as chlorotic interveinal yellow patches of the older leaves (**Figure 1**). The severity of symptoms increased with time, depending on the form and concentration of the iodine applied as well as on the cultivar. In KI-treated plants (**Figure 1**), early phytotoxicity symptoms appeared already after a few days as shorten new developed nodes, discolorations and chlorotic areas in the leaves. After 2 weeks iodine treatment, the plants showed serious phytotoxicity symptoms on all the leaves and few bottom leaves had also dropped.

**Figure 2:**
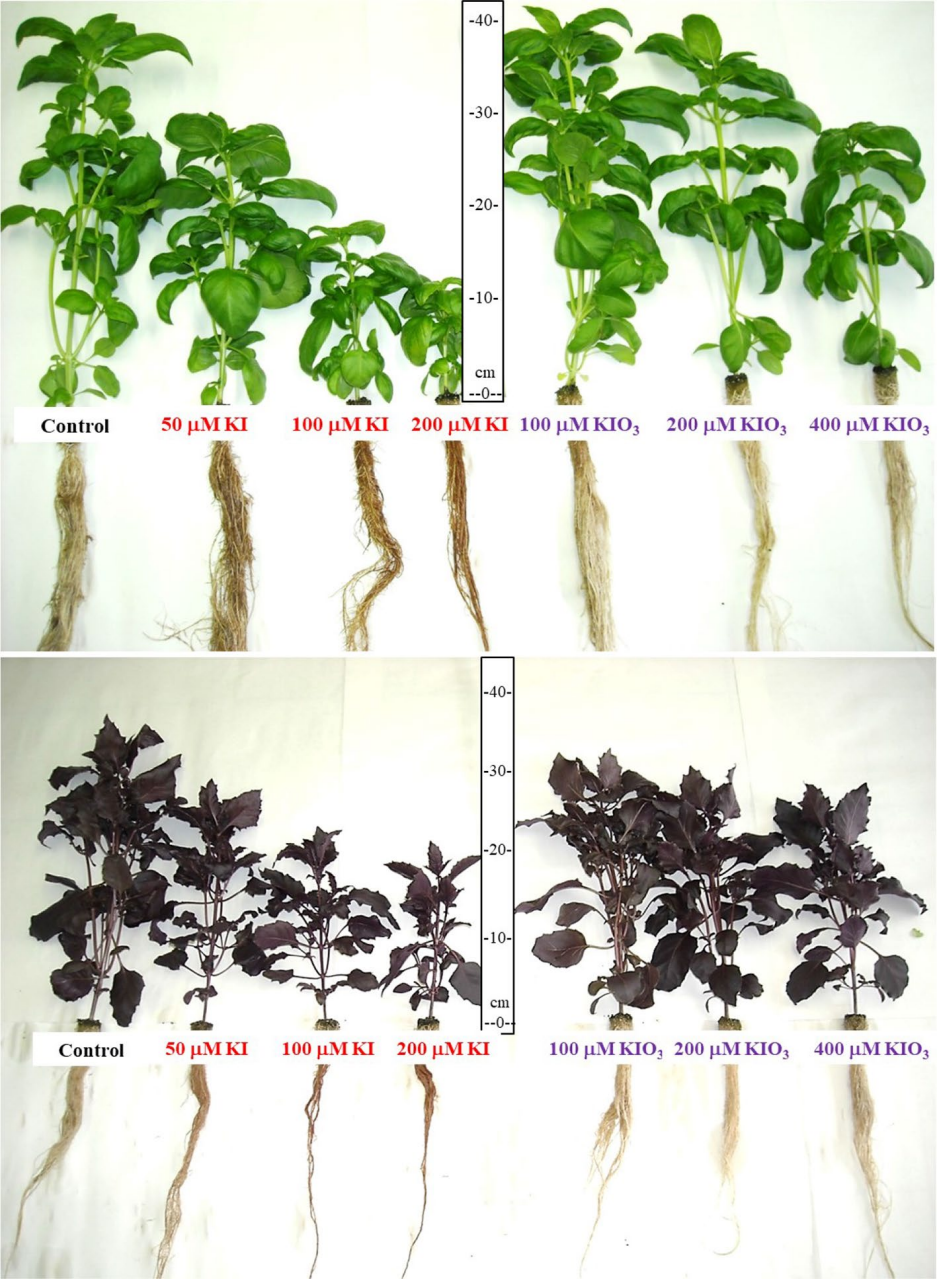
Effect of increasing concentrations of potassium iodide (KI; control = 0.1 μM) or potassium iodate (KIO_3_) in the nutrient solution on the growth of two cultivars (“Tigullio,” top and “Red Rubin,” bottom) of sweet basil (*Ocimum basilicum* L.). For each cultivar and each iodine concentration, both the aerial part and the root system of the plants at the end of the experiment are shown. Plants were grown in floating system for 14 days, from 9^th^ to 23^rd^ May 2016 (*Experiment 3*).

The symptoms were less severe in “Red Rubin” than in “Tigullio” and when the plants were treated with KIO_3_ than with KI at the same concentrations (**Figure 2**).

### Experiment 1

The green-leaved cultivar “Tigullio” grew faster than “Red Rubin” and after 21 days the total plant dry weight of “Tigullio” plants was approximately three times higher than the dry weight of “Red Rubin” plants.

Neither 10 nor 100 µM KIO3 concentrations affected plant growth in both cultivars. On the contrary, 100 µM KI in the nutrient solution significantly reduced the dry weight of roots, leaves and stems, respectively, by 22, 67, and 80% in “Tigullio,” and by 17, 22, and 23% in “Red Rubin.” When the plants were exposed to 100 µM KI, plant height and leaf area were reduced, respectively, by 64 and 68% in “Tigullio” and by 33 and 13% in “Red Rubin” (**Table SM1**).

### Experiment 2

After 16 days treatment, a reduction in plant growth was observed in both basil cultivars at KI concentrations higher than 10 µM; however, growth inhibition was less severe in “Red Rubin” than in “Tigullio” (**Table 2**). For example, compared to the control, 200 µM KI concentration reduced total plant dry matter and plant height, respectively, by 42 and 46% in “Red Rubin,” and both by 73% in “Tigullio”. In both cultivars, leaf iodine content increased with increasing KI concentration in the nutrient solution (**Table 2**). Iodine accumulation in leaf tissues was more pronounced in “Red Rubin” than in “Tigullio”: at 200 µM KI concentration, leaf iodine contents were 6,955 ± 915 in “Red Rubin” and 5,225 ± 380 mg kg^−1^ DW in “Tigullio.”

**Table 2:** Effect of different concentrations of potassium iodide (KI; control = 0.1 μM) in the nutrient solution on plant height, root, stem, leaves and total dry weight (DW), and leaf iodine content of two cultivars (“Tigullio” and “Red Rubin”) of sweet basil (*Ocimum basilicum L*.).Each value is the mean (± standard deviation, SD) of three replicates. Plants were grown hydroponically under greenhouse conditions for 16 days from 29^th^ June to 14^th^ July 2015 (Experiment 2).

Cultivar	Treatment	Plant height (cm plant^−1^)	Root DW (g plant^−1^)	Stem DW (g plant^−1^)	Leaf DW (g plant^−1^)	Total DW (g plant^−1^)	Leaf iodine content (mg kg^−1^)
“Tigullio”	Control	41.5 ± 2.0 a	1.05 ± 0.06 a	3.05 ± 0.35 a	5.43 ± 0.32 a	9.53 ± 0.62 a	23 ± 5g
	10 μM KI	40.7 ± 0.7 a	1.00 ± 0.07 a	3.17 ± 0.23 a	5.51 ± 0.46 a	9.68 ± 0.64 a	295 ± 48 f
	50 μM KI	35.4 ± 1.4 b	0.89 ± 0.07 b	2.27 ± 0.22 b	4.55 ± 0.21 b	7.71 ± 0.42 b	1423 ± 155 e
	100 μM KI	21.2 ± 1,1 e	0.61 ± 0.08 c	1.15 ± 0.15 d	2.87 ± 0.17 ef	4.63 ± 0.38 e	3093 ± 311 d
	200 μM KI	11.3 ± 0.9 g	0.49 ± 0.05 d	0.37 ± 0.08 f	1.69 ± 0.21 g	2.54 ± 0.31 g	5225 ± 380 b
“Red Rubin”	Control	31.1 ± 1.9 c	0.49 ± 0.03 d	1.79 ± 0.18 c	3.45 ± 0.09 cd	5.73 ± 0.30 c	25 ± 6g
	10 μM KI	29.6 ± 0.6 cd	0.46 ± 0.03 de	1.66 ± 0.24 c	3.62 ± 0.16 c	5.74 ± 0.24 c	420 ± 29 f
	50 μM KI	27.8 ± 0.2 d	0.40 ± 0.04 e	1.23 ± 0.09 d	3.39 ± 0.14 d	5.02 ± 0.08 d	1645 ± 121 e
	100 μM KI	21.3 ± 1.2 e	0.39 ± 0.05 e	1.06 ± 0.16 e	3.04 ± 0.09 e	4.49 ± 0.18 e	3865 ± 404 c
	200 μM KI	16.1 ± 1.1 f	0.31 ± 0.04 f	0.47 ± 0.10 f	2.53 ± 0.07 f	3.31 ± 0.19 f	6955 ± 915 a
**Analysis of variance**							
Cultivar		***	***	***	***	***	***
Treatment		***	***	***	***	***	***
Cultivar×Treatment		***	***	***	***	***	***

Significance: *** P < 0.001. Values followed by the same letter within a column are not significantly different (P < 0.05; Duncan’s test).

The Maas-Hoffman equations for relative plant height or dry biomass against the KI concentration in the nutrient solution are reported in **Table 3** and **Figure SM1**. In “Tigullio” the threshold iodine concentrations (*t*) for leaf DW, total plant DW and plant height were within 17.8 µM and 12.4 µM (on average, *t* was 16.0 µM), while the slope *s* was similar for the three growing parameters under consideration (on average, *s* was −0.42% µM^−1^). Compared with “Tigullio,” “Red Rubin” showed a higher *t* (on average, it was 29.8 µM) and a lower absolute value of *s* (−0.24% µM^−1^, on average).

**Table 3:** Maas-Hoffman equation for relative (Y*, %) plant height and relative leaf and total dry weight against the concentration of potassium iodide (X = 0.1, 10, 50, 100 and 200 μM) in the nutrient solution or leaf iodine content (mg kg^−1^ DW) of two cultivars (“Tigullio” and “Red Rubin”) of sweet basil (*Ocimum basilicum* L.). Plants were grown hydroponically under greenhouse conditions for 16 days from 29^th^ June to 14^th^ July 2015 (*Experiment 2*).

Parameter	Maas-Hoffman equation	R^2^	Maas-Hoffman equation	R^2^
	Tigullio		Red Rubin	
**Nutrient solution iodine concentration (µM)**				
Plant height	Y* = 100−0.43 (X−17.8)	0.87	Y* = 100−0.30 (X−26.7)	0.85
Leaves	Y* = 100−0.40 (X−12.4)	0.84	Y* = 100−0.18 (X−33.4)	0.92
Whole plant	Y* = 100−0.45 (X−17.8)	0.84	Y* = 100−0.24 (X−29.2)	0.81
**Leaf iodine concentration (mg kg^−1^ DW)**				
Leaves	Y* = 100−0.015 (X−272)	0.96	Y* = 100−0.005 (X−667)	0.92

When plant growth response was analysed against leaf iodine concentrations, *t* and *s* were, respectively, 272 mg kg^−1^ DW and −0.015% mg^−1^ kg DW in “Tigullio,” and 667 mg kg^−1^ DW and −0.005% mg^−1^ kg DW in “Red Rubin” (**Table 3**).

### Experiment 3

The main goal of this experiment was to determine if the growth inhibition of sweet basil due to toxic iodine concentrations in the nutrient solution was associated with a reduced uptake of other nutrients. Different concentrations of KI (50, 100, and 200 µM) or KIO_3_ (100, 200, and 400 µM) were tested in order to induce different levels of growth inhibition.

As found in *Experiments 1* and *2*, “Red Rubin” showed a higher iodine tolerance than “Tigullio” and KI was more toxic than KIO_3_ (Supplementary Material, **Table SM2**). The growth reduction induced by 400 µM KIO_3_ was similar to the effect produced by the 50 µM KI treatment (**Figure 2**; Supplementary Material, **Table SM2**).

In the control, there was no important difference between “Tigullio” and “Red Rubin” as regards the leaf concentration of macronutrients and micronutrients, apart from a slightly lower content of P in “Red Rubin” (**Table 4**).

**Table 4:** Effect of different concentrations of potassium iodide (KI; control = 0.1 μM) or potassium iodate (KIO_3_) in the nutrient solution on some leaf mineral content (N–NO_3_, total N, P, K, Ca, Mg, Fe, Mn, Zn, and Cu) of two cultivars (“Tigullio” and “Red Rubin”) of sweet basil (*Ocimum basilicum* L.). Each value is the mean (± SD) of three replicates. Plants were grown hydroponically under greenhouse conditions for 14 days from 9^th^ to 23^rd^ May 2016 (Experiment 3).

Treatment	NO_3_^-^ (mg kg^−1^ FW)	N (%)	P (%)	K (%)	Ca (%)	Mg (%)	Fe (mg kg^−1^)	Mn (mg kg^−1^)	Zn (mg kg^−1^)	Cu (mg kg^−1^)
Tig. Control	1196 ± 85 e	4.23 ± 0.22 a	0.82 ± 0.09 a	5.72 ± 0.08 a	2.13 ± 0.12 d	0.43 ± 0.02	101 ± 15 c	83 ± 8	123 ± 10 b	13 ± 2
Tig. 50 μM KI	1229 ± 72 e	4.20 ± 0.16 a	0.73 ± 0.06 ab	5.54 ± 0.15 ab	2.28 ± 0.10 c	0.41 ± 0.02	108 ± 10 c	78 ± 10	81 ± 11 c	13 ± 1
Tig. 100 μM KI	1362 ± 99 de	3.92 ± 0.26 ab	0.65 ± 0.06 b	5.29 ± 0.51 b	2.13 ± 0.12cd	0.41 ± 0.03	114 ± 11 c	71 ± 10	63 ± 8 c	12 ± 2
Tig. 200 μM KI	1661 ± 65 bc	3.32 ± 0.15 b	0.60 ± 0.07 bc	4.67 ± 0.39 c	2.55 ± 0.28 b	0.42 ± 0.06	141± 21b	78 ± 7	86 ± 8 c	13 ± 1
Tig. 100 μM KIO_3_	1329 ± 70 de	4.57 ± 0.17 a	0.84 ± 0.11 a	5.80 ± 0.40 a	2.57 ± 0.12 b	0.43 ± 0.03	135 ± 18 b	87 ± 10	139 ± 11 ab	15 ± 2
Tig. 200 μM KIO_3_	1362 ± 76 de	4.51 ± 0.11 a	0.84 ± 0.07 a	5.85 ± 0.15 a	2.47 ± 0.11 b	0.39 ± 0.03	126 ± 13 b	91 ± 9	154 ± 12 a	14 ± 2
Tig. 400 μM KIO_3_	1461 ± 83 d	3.97 ± 0.20 ab	0.86 ± 0.16 a	5.48 ± 0.31 ab	2.91 ± 0.19 a	0.42 ± 0.05	135 ± 12 b	90 ± 12	149 ± 10 a	14 ± 2
RR. Control	1627 ± 66 bc	4.27 ± 0.08 a	0.69 ± 0.10 b	5.50 ± 0.73 ab	2.04 ± 0.15 d	0.40 ± 0.03	110 ± 20 c	83 ± 9	108 ± 17 bc	16 ± 2
RR. 50 μM KI	1794 ± 100 ab	3.98 ± 0.20 ab	0.64 ± 0.05 b	5.12 ± 0.29 b	2.17 ± 0.24 cd	0.41 ± 0.02	159 ± 22 a	75 ± 10	83 ± 8 c	15 ± 1
RR. 100 μM KI	1827 ± 63 a	4.05 ± 0.07 a	0.58 ± 0.06 c	5.03 ± 0.18 bc	2.00 ± 0.12 d	0.39 ± 0.02	147 ± 25 ab	65 ± 9	58 ± 9 c	14 ± 1
RR. 200 μM KI	1860 ± 85 a	3.25 ± 1.17 b	0.57 ± 0.05 c	4.87 ± 0.35 c	2.16 ± 0.21 c	0.44 ± 0.04	135 ± 18 b	74 ± 11	68 ± 7 c	13 ± 1
RR. 100 μM KIO_3_	1727 ± 66 b	4.52 ± 0.11 a	0.68 ± 0.09 b	5.04 ± 0.24 bc	2.27 ± 0.24 c	0.41 ± 0.03	159 ± 10 a	77 ± 12	138 ± 12 a	17 ± 2
RR. 200 μM KIO_3_	1794 ± 78 ab	4.48 ± 0.10 a	0.69 ± 0.07 b	5.35 ± 0.15 ab	2.24 ± 0.13 c	0.39 ± 0.02	160 ± 15 a	83 ± 6	154 ± 13 a	16 ± 2
RR. 400 μM KIO_3_	1845 ± 91 a	4.44 ± 0.08 a	0.67 ± 0.06 b	5.18 ± 0.38 b	2.89 ± 0.20 a	0.37 ± 0.04	128 ± 11 b	80 ± 11	160 ± 14 a	12 ± 1
**Analysis of variance**										
Cultivar	*	ns	***	ns	ns	ns	ns	ns	ns	ns
Treatment	**	***	***	**	*	ns	**	ns	**	ns
Cultivar×Treatment	ns	*	Ns	*	**	ns	ns	ns	ns	ns

Significance: * P < 0.05; ** P < 0.01; *** P < 0.001. ns: not significant. Values followed by the same letter within a column are not significantly different (P < 0.05; Duncan’s test).

Iodine level did not influence the leaf concentrations of Mg (0.41% ± 0.04 mg kg^−1^ DW), Mn (77 ± 12 mg kg^−1^ DW) and Cu (14.1 ± 2.5 mg kg^−1^ DW). Compared with the control, the 200 µM KI treatment decreased the leaf content of total N, P, K, and Zn, and increased the leaf content of Ca and Fe in both basil cultivars (**Table 4**). Evident effects of KIO_3_ on leaf nutrient content were observed only in the 400 µM treatment in both cultivars, and consisted in a significant increase in the leaf content of Ca, Fe and Zn, without any significant reduction in the content of other nutrients.

The addition of iodine to the nutrient solution did not have important effects of leaf nitrate content (expressed on a fresh weight basis). Compared to the control (1,196 ± 85 mg NO_3_^−^ kg^−1^ FW) a significant increase in leaf nitrate content was observed only in “Tigullio” plants grown at 200 µM KI (1,661 ± 65 mg NO_3_^−^ kg^−1^ FW). In general, “Red Rubin” accumulated more nitrates in leaf tissues than “Tigullio.”

**Figure 3:**
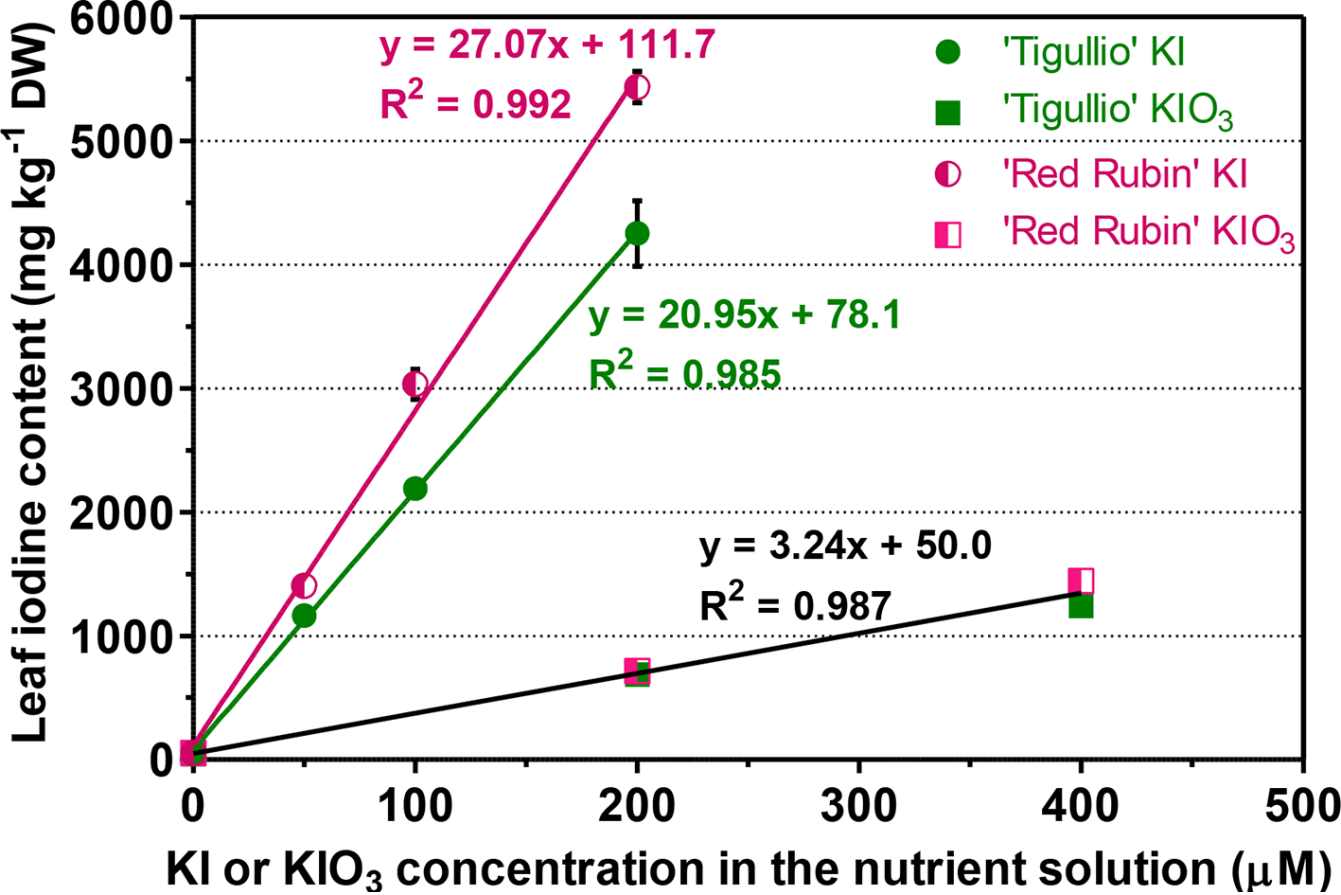
Influence of different concentration of potassium iodide (KI; control = 0.1 μM) or potassium iodate (KIO_3_) in the nutrient solution on the iodine concentration in the leaf tissues of two cultivars (“Tigullio” and “Red Rubin”) of sweet basil (*Ocimum basilicum* L.). Each value is the mean (± SD) of three replicates. The linear regression lines for the KI treatments are reported along with the equations for both varieties. For the KIO_3_ treatments, only the result of the regression analysis of the pooled data of both cultivars is reported, since the individual slopes and intercepts for each cultivar are not significantly different *(P < 0.05*). Plants were grown hydroponically under greenhouse conditions for 14 days from 9^th^ to 23^rd^ May 2016 (*Experiment 3*).

At the end of the experiment, in both the cultivars and for all the concentrations tested, KI produced a greater increase in iodine leaf content than the same concentration of KIO_3_ (**Figure 3**). For example, a 14-days cultivation in a nutrient solution containing 200 µM KI or KIO_3_ produced an iodine leaf content of 4,250 ± 460 or 687 ± 84 mg kg^−1^ DW in “Tigullio” and 5,433 ± 218 or 717 ± 68 mg kg^−1^ DW in “Red Rubin,” respectively. A significant linear relationship between iodine leaf content and KI or KIO_3_ concentration in the nutrient solution was found for both cultivars. However, for the KIO_3_ treatments, the slopes and intercepts of the regression lines for “Tigullio” and ‘Red Rubin’ were not significantly different (P < 0.05); therefore, the data of both cultivars were pooled and a single regression line was obtained (**Figure 3**).

### Experiment 4

In this experiment we investigated the relationship between CO_2_ assimilation (A) and iodine content in plants of both cultivars grown with different concentrations of KI (50, 100 and 200 µM) or KIO_3_ (100, 200 and 400 µM). The determinations were performed after 12 days of treatment on basal leaves (2^nd^ node) that were already present at the start of the experiment.

Both 100 and 200 µM KI caused a significant decrease of dry biomass accumulation and leaf area, while only 200 µM KIO_3_ induced a slight reduction of these parameters (data not shown) in agreement with the results of the previous experiments. For the sake of brevity, only the mean values of the total leaf area at the end of the experiment are here reported, for “Tigullio” and “Red Rubin”: 906 ± 64 and 602 ± 55, 897 ± 50 and 564 ± 48, 824 ± 42 and 445 ± 37, 499 ± 51 and 426 ± 29, 376 ± 32 and 401 ± 21 cm^2^ plant^−1^ for control, 100 µM KIO_3_, 200 µM KIO_3_, 100 µM KI, and 200 µM KI treatments, respectively.

**Figure 4:**
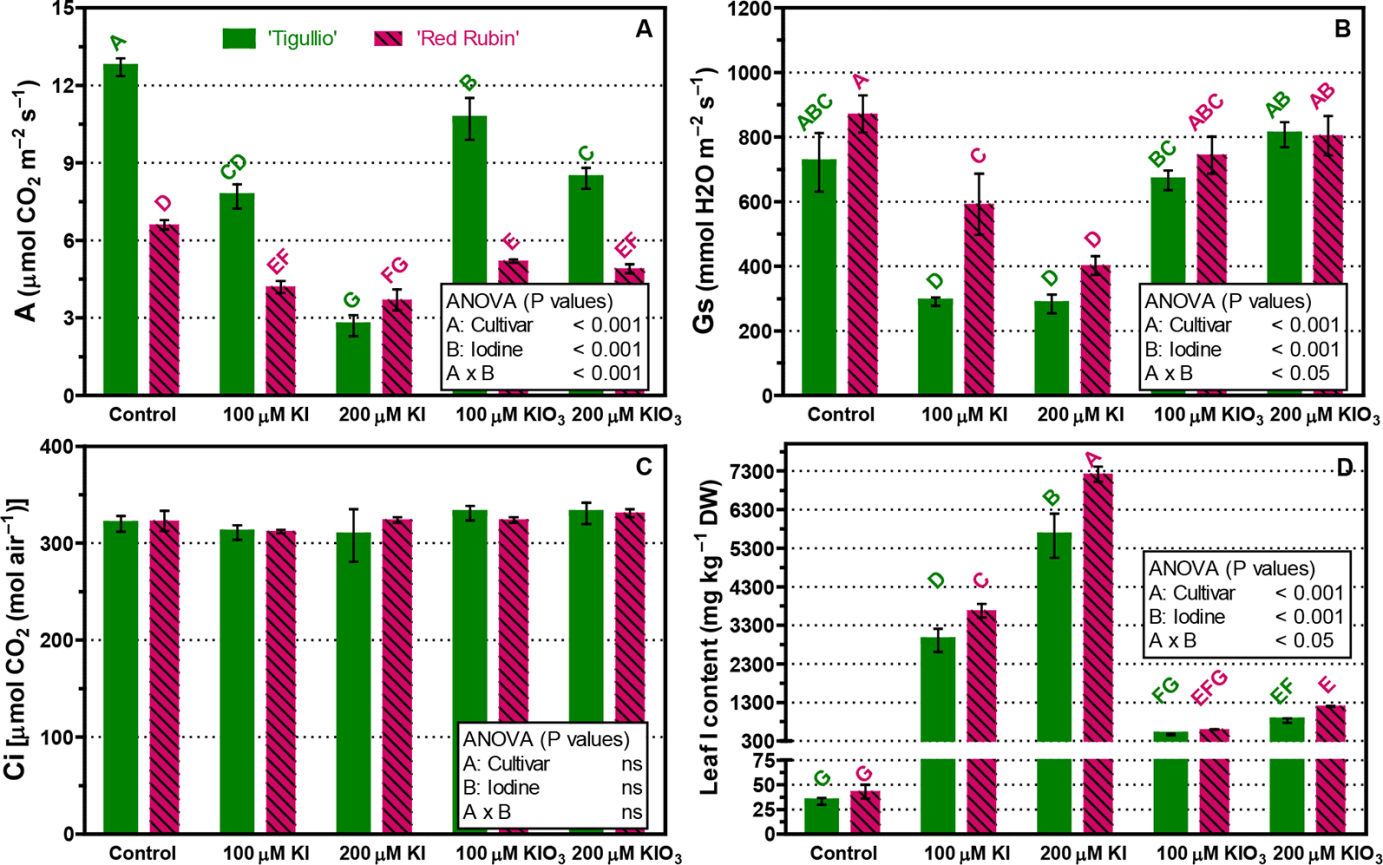
Influence of different concentrations of potassium iodide (KI; control = 0.1 μM) or potassium iodate KIO_3_ in the nutrient solution on net assimilation (A), stomatal conductance (Gs), internal CO_2_ concentration (Ci) and iodine content of basal leaves of two cultivars (“Tigullio” and “Red Rubin”) of sweet basil (*Ocimum basilicum* L.). The measurements were taken on the opposite leaves of the 2^nd^ node, at the end of the experimental period. Each value is the mean of three replicates (± S.D.); bars with the same letter indicate values that are not significantly different (*P < 0.05*). Plants were grown hydroponically under greenhouse conditions for 12 days from 9^th^ to 21^st^ May 2016 (*Experiment 4*).

In the control, net photosynthesis (A) was approximately 50% lower in “Red Rubin” than in “Tigullio” (**Figure 4A**). In both basil cultivars, all tested KI and KIO_3_ concentrations significantly decreased leaf photosynthesis (**Figure 4A**) while stomatal conductance (Gs) was significantly reduced only by KI (**Figure 4B**). In “Tigullio” plants, no significant effects of iodine were found on internal CO_2_ concentrations (Ci; **Figure 4C**).

In general, the basal leaves of “Red Rubin” had a higher iodine content than those of “Tigullio” (**Figure 4D**). For example, at 200 µM KI leaf iodine content was 5,623 ± 995 mg kg^−1^ DW in “Tigullio” and 7,720 ± 346 mg kg^−1^ DW in “Red Rubin” (**Figure 4D**).

**Figure 5:**
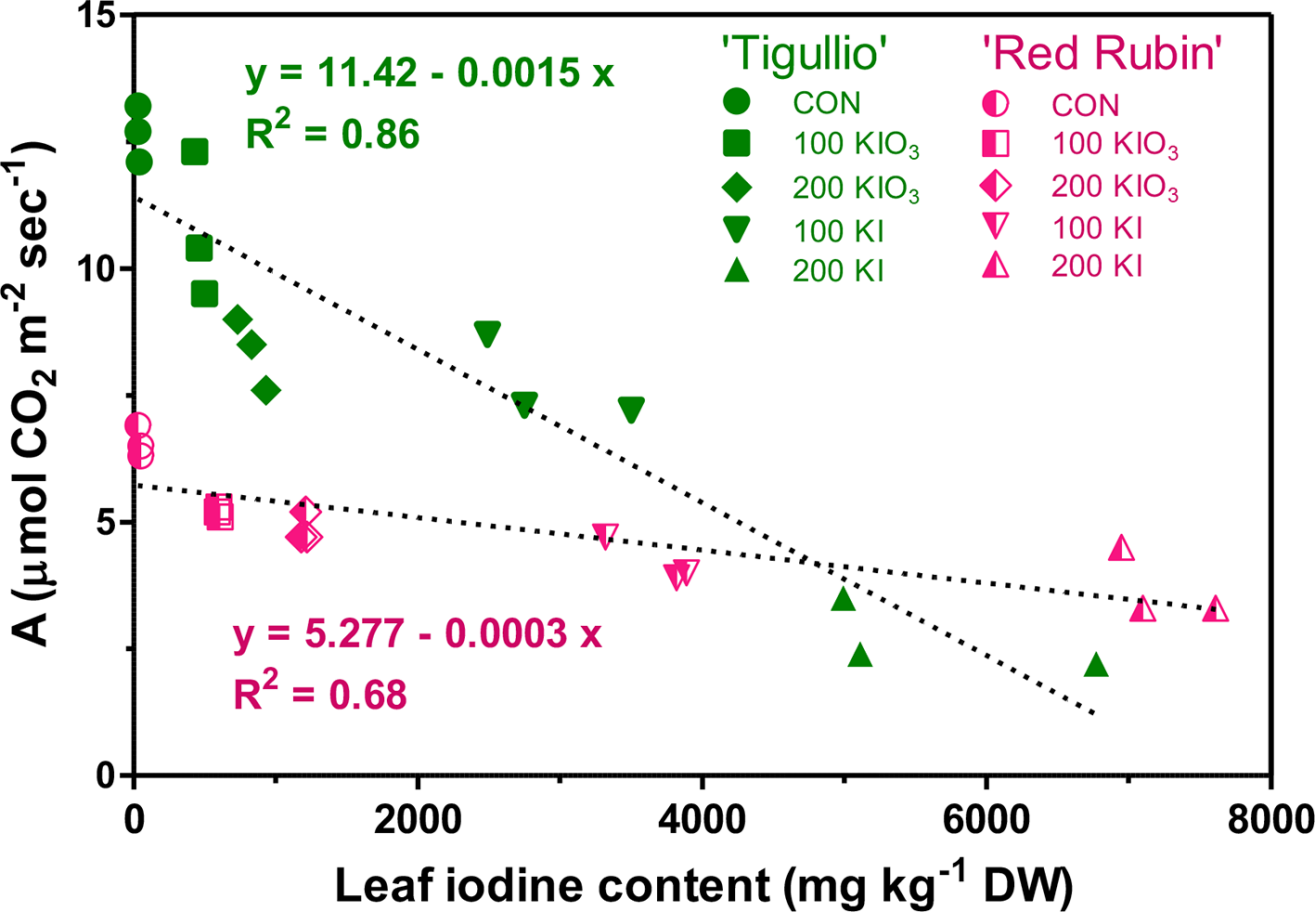
Linear regression between the net assimilation (A) and iodine in the basal leaves of two cultivars (“Tigullio” and “Red Rubin”) of sweet basil (*Ocimum basilicum* L.) grown hydroponically with different concentrations of potassium iodide (KI; control = 0.1 μM) or potassium iodate (KIO_3_) in the nutrient solution. The measurements were taken on the opposite leaves of the 2^nd^ node at the end of the experimental period. Plants were grown hydroponically under greenhouse conditions for 12 days from 9^th^ to 21^st^ May 2016 (*Experiment 4*).

In both basil cultivars, a close negative correlation was found between A and iodine content as measured on the same leaves, with significant differences between “Tigullio” and “Red Rubin” as regards the intercept and the slope of the linear regression (**Figure 5**).

**Figure 6:**
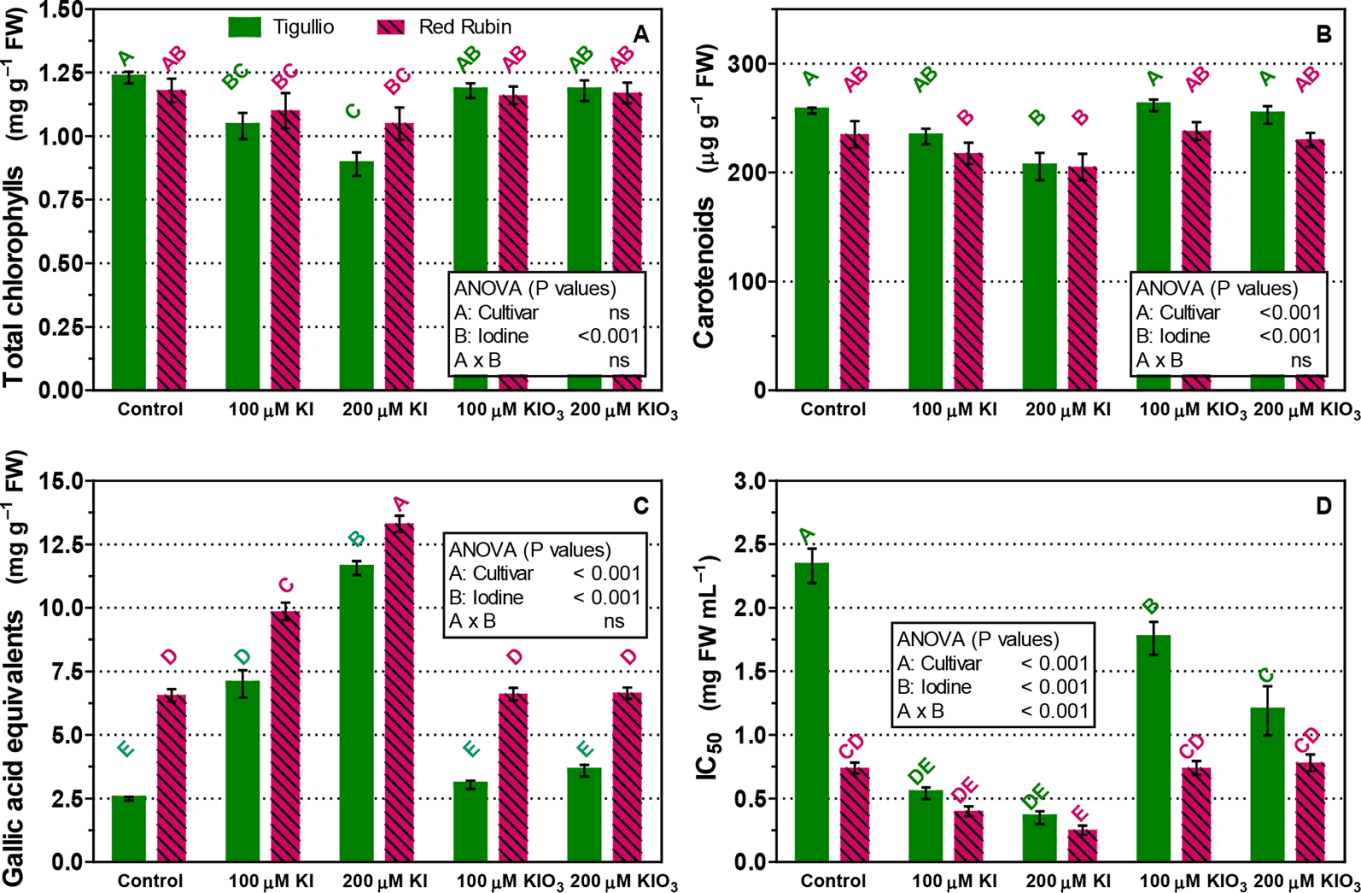
Influence of the concentration of potassium iodide (KI; control = 0.1 μM) or potassium iodate (KIO_3_) in the nutrient solution on the concentration of total chlorophylls **(A)**, carotenoids **(B)**, total phenols as gallic acid equivalents **(C)** and antioxidant capacity (IC_50_) **(D)** in the basal leaves of two cultivars (“Tigullio” and “Red Rubin”) of sweet basil (*Ocimum basilicum* L.). The measurements were taken at the opposite leaves of the 2^nd^ node at the end of the experimental period. Each value is the mean of four replicates (± SD); bars with the same letter indicate values that are not significantly different (*P* < 0.05). Plants were grown hydroponically under greenhouse conditions for 12 days from 9^th^ to 21^st^ May 2016 (*Experiment 4*).

The content of carotenoids, total chlorophylls and phenols (gallic acid equivalents), and total antioxidant capacity were also determined in the same leaves sampled for leaf gas exchange measurements (**Figure 6**). In “Tigullio” a reduced content of total chlorophylls and carotenoids compared with the control (1.23 ± 0.04 and 0.26 ± 0.01 mg g^−1^ FW, respectively) was observed with 100 and 200 µM KI (on average, 0.96 ± 0.8 and 0.22 ± 0.02 mg g^−1^ FW, respectively), while no significant effect was observed in the leaf pigment concentrations of “Red Rubin” (1.13 ± 0.10 and 0.23 ± 0.02 mg g^−1^ FW, for total chlorophylls and carotenoids, respectively; **Figures 6A, B**). Moreover, the A reduction observed in “Tigullio” with the 200 µM KI treatment was associated to a significantly lower concentration of carotenoids and total chlorophylls (**Figures 6A, B**).

Both 100 and 200 µM KI treatments determined an increase in total phenols and antioxidant capacity on mature leaves (2^nd^ node, **Figures 6C, D**). In both cultivars, the concentration of total phenols increased with the KI concentration in the nutrient solution and was not influenced by KIO_3_. In the control, both the concentration of total phenols and the antioxidant capacity (DPPH assay) were much higher in “Red Rubin” than in “Tigullio” (**Figures 6C, D**): 6.56 ± 0.48 against 2.49 ± 0.04 mg g^−1^ FW of gallic acid equivalents, and 0.74 ± 0.09 and 2.33 ± 0.27 mg FW ml^−1^, respectively for the inhibitory concentration (IC_50_). In addition to the DPPH assay, we performed also the FRAP assay on our samples: the data of the two methods showed a very good correlation for both “Tigullio” and “Red Rubin” (Pearson’s coefficients 0.994 and 0.892, respectively).

**Figure 7:**
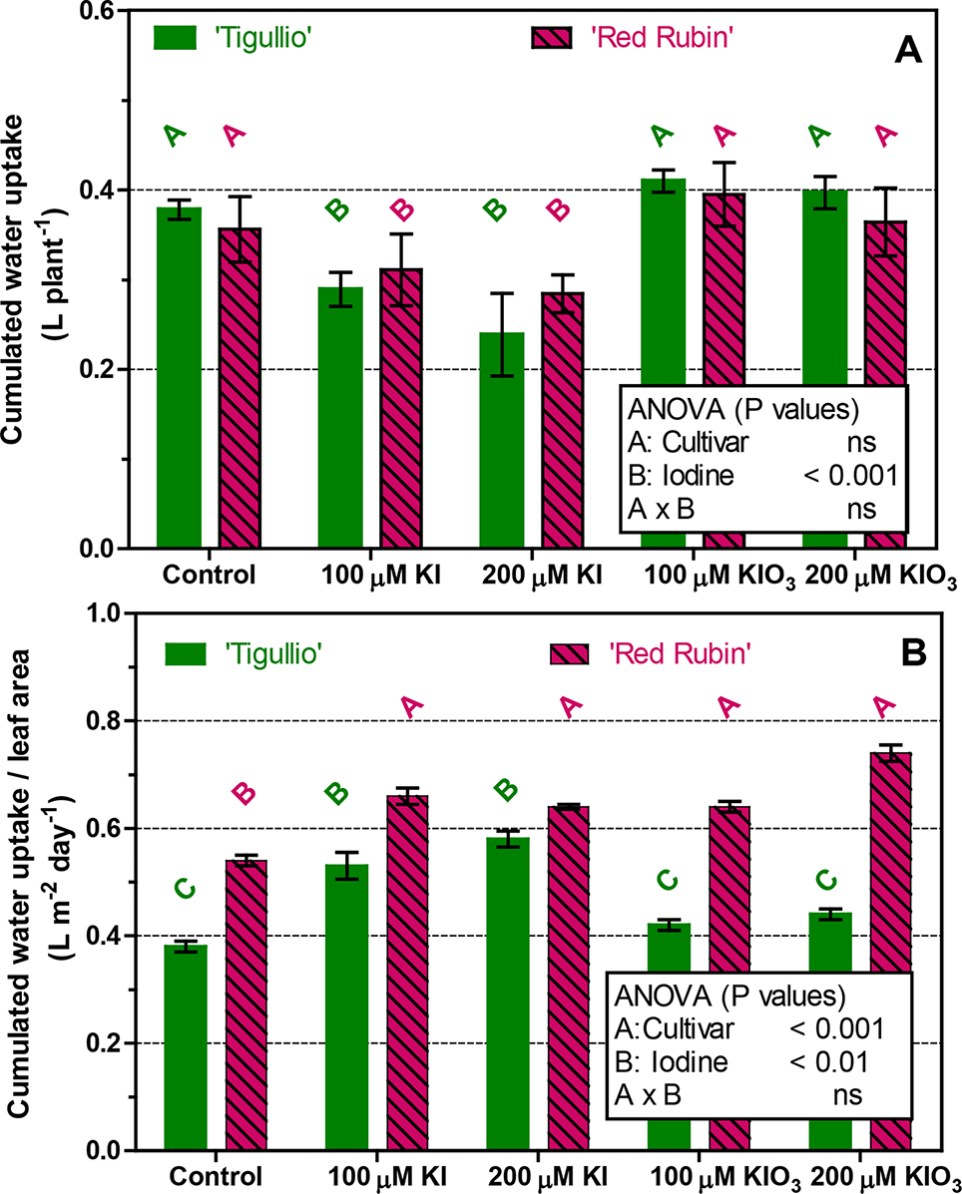
Influence of different concentrations of potassium iodide (KI; control = 0.1 μM) or potassium iodate (KIO_3_) in the nutrient solution on the cumulated water uptake (expressed per plant or on leaf area basis) of two cultivars (“Tigullio” and “Red Rubin”) of sweet basil (*Ocimum basilicum* L.). Each bar is the mean (± SD) of four replicates; bars with the same letter indicate values that are not significantly different (*P* < 0.05). Plants were grown hydroponically under greenhouse conditions for 12 days from 9^th^ to 21^st^ May 2016 (*Experiment 4*).

The cumulated water uptake during *Experiment 4* was also measured (**Figure 7A**). In the control, water uptake was similar in the two cultivars when expressed per plant (**Figure 7A**), while it was significantly higher in “Red Rubin” when expressed on a leaf area basis (**Figure 7B**). For both cultivars, no differences were found among the control and 100 and 200 µM KIO_3_. Compared to the control, whole plant water uptake was significantly decreased at 100 and 200 µM KI, while an opposite result was found when water uptake was expressed on a leaf area basis (**Figure 7B**).

## Discussion

### Effects of Iodine on Plant Growth

In this work, KI and KIO_3_ concentrations not higher than 10 µM or 100 µM, respectively, did not influence the total biomass in “Tigullio” or “Red Rubin” cultivars (**Table 2**; **Tables SM1** and **SM2**). Nevertheless, the use of nutrient solution with 50 µM KI or 400 µM KIO_3_ resulted in a similar reduction of plant growth (**Table SM2**), thus suggesting that in sweet basil I^−^ added to the nutrient solution is approximately eight-times more phytotoxic than IO_3_^−^.

In both cultivars, higher concentrations of KI (100 or 200 µM) in the nutrient solution caused a significant decrease of plant height, total dry weight and leaf area compared to the control, and growth inhibition was more severe in “Tigullio” than in “Red Rubin” (**Table 2**; **Table SM2**), suggesting a higher iodine tolerance of the red-leaved cultivar. This conclusion was corroborated by the result of the application of the Maas-Hoffman model to assess the plant response to iodine supply, as a lower slope and a higher critical concentration (the threshold) was computed for “Red Rubin” than for “Tigullio” (**Table 3**).

Several authors found a positive effect of iodine applied to the nutrient solution on plant growth, especially if this element was supplied at low concentration and/or as IO_3_^−^ (Borst Pauwels, 1961 on barley and tomato; Zhu et al., 2003 on spinach; Li et al., 2017 on strawberry). For example, Li et al. (2017) reported an increase of plant biomass in strawberry when iodine was applied to the nutrient solution up to 1.97 µM as I^−^ and 2.86 µM as IO_3_^−^. In contrast, detrimental effects were observed when the I^−^ or IO_3_^−^ concentrations in the nutrient solution were higher than 10–40 µM or 100–200 µM, respectively (Blasco et al., 2008). In our experiments, the addition of KI or KIO_3_ to the nutrient solution never produced a significant increase of total biomass with respect to the control, probably due to the much higher concentrations (10 µM or higher) tested in comparison to those reported in the literature.

A decrease in the biomass of iodine treated plants was also reported in lettuce (Blasco et al., 2012; Smoleń et al., 2014a), tomato and potato (Caffagni et al., 2011), as well as in carrot (Smoleń et al., 2014b) and in *Opuntia* (García-Osuna et al., 2014), although in other plant species whose vegetative reserve organs are also harvested, such as onion, iodine seemed to have no effect on plant weight (Dai et al., 2009).

### Effects of Iodine on Leaf Photosynthesis and Water Uptake

Toxic iodine concentrations in the nutrient solution significantly reduced the CO_2_ assimilation of mature leaves in both basil cultivars, albeit to a lesser extent in “Red Rubin,” which had a lower photosynthetic capacity compared to “Tigullio” (**Figure 4A**). In both cultivars, leaf CO_2_ assimilation was negatively correlated to leaf iodine content (**Figure 5**); however, the slope of the regression line between these parameters was much lower in “Red Rubin” than in “Tigullio” and this is a further evidence of the greater iodine tolerance of the purple-leaved cultivar. In “Tigullio,” the reduction of CO_2_ assimilation was attributable to both stomatal and mesophilic limitations as leaf stomatal conductance decreased while the intercellular CO_2_ concentrations did not change following iodine stress (**Figure 4C**). On the contrary, only mesophilic limitations were observed in “Red Rubin.” Application of 80 µM KI reduced leaf CO_2_ assimilation in lettuce and this effect was associated with a reduction of stomatal conductance and total chlorophyll (Blasco et al., 2011).

In “Tigullio” the detrimental effects of 100 and 200 µM KI on leaf CO_2_ assimilation were associated to a reduced content of total chlorophylls and carotenoids (**Figures 6A, B**), which was also revealed by the occurrence of leaf chlorosis. No significant effects of iodine level were found on the leaf pigment concentrations of “Red Rubin.”

The reduction of the content of photosynthetic pigments due to toxic concentration of KI was found in barley (Duborská et al., 2018) and in lettuce (Lawson et al, 2015; Medrano-Macías et al., 2016).

The reduction of stomatal conductance observed in mature leaves of “Tigullio” following KI application was not associated with reduced water uptake as expressed on a leaf area basis, which instead increased in both cultivars (**Figure 7**). The cumulated water uptake of whole plants, which was accounted for 94–96% by transpiration in previous experiments with the same sweet basil cultivars (Pardossi et al., 2015), was decreased by KI application as a result of reduced leaf area.

In all the treatments, “Red Rubin” showed a higher water uptake per leaf area rate than “Tigullio” (**Figure 7B**), probably due to the purple colour of leaves that resulted in a higher average leaf temperature (0.6–1.0°C more than “Tigullio,” data not shown), and thus favoured the transpiration process.

Accordingly, the experiments of Voogt et al. (2010; 2014) on the iodine biofortification of lettuce, cucumber, pepper, round and cherry tomato highlighted that the application of iodine did not alter the water uptake capacity of the crop, and possibly the cumulated water uptake differences could be ascribed to the total leaf area reduction.

### Effects of Iodine on Nutrient Uptake

The growth reduction observed in both cultivars exposed to 100 and 200 µM KI was associated with a significant reduction of the leaf concentration of N, P, K (not in “Red Rubin”), and Zn (**Table 4**). Leaf contents of Mg, Mn, and Cu were not affected, while the contents of Fe and Ca were significantly increased. The application of KI also increased the leaf concentration of nitrate.

Similar results were found in lettuce grown in soilless culture by Blasco et al. (2012). These authors found that, compared to the control, the application of 40 µM KI decreased the leaf content of N, P, and K and increased Fe content without significant effects on the leaf concentration of Cu and Mn.

Low iodine fertilizer concentrations (100 µM KI or KIO_3_) added in the irrigation water were found to increase P, Mg, Fe, K, Cu, and Mn levels in *Opuntia Ficus*-*indica* (García-Osuna et al., 2014).

In contrast with our findings, Smoleń et al. (2015a) found a negative correlation in field-grown lettuce between the concentration of iodine and those of Mg, Ca, S, Na, B, Cu, Fe, Mn, Zn, and K (the latter in agreement with our results).

Blasco et al. (2012) suggested that high concentrations of iodine may alter the specific root transporters for NO_3_^−^, affect P and K uptake or trigger an antagonistic interaction that would explain the reduction of these elements in the leaves of basil plants. Schlorke et al. (2016) suggested that a high iodine content in plant tissues could induce detoxifying mechanisms that increase the activity of oxidizing enzymes and lead to changes in tissue concentrations of Cu, Fe, and Mn. Alternatively, Kato et al. (2013) suggested that IO_3_^−^ may induce reductase activity in the root, which could impact on the bioavailability of mineral nutrients, and that the activation of iodate reductase could induce other responses associated with redox signalling to counteract the effect of IO_3_^−^.

Indeed, in our work the leaf concentration of all considered nutrients remained within the sufficiency ranges reported for sweet basil (Zheljazkov and Warman, 2003); therefore, the remarkable growth reduction observed in plants treated with KI cannot be explained by iodine-induced mineral deficiencies. Similar conclusions were reported for barley by Duborská et al. (2018) and for lettuce by Blasco et al. (2012) and Smoleń et al. (2014a).

Sweet basil is a vegetable that can accumulate high content of nitrates (Muráriková and Neugebauerová, 2018). The application of KI also increased the leaf concentration of NO_3_^−^ (**Table 4**). An increase in leaf NO_3_^−^ content due to iodine exposure was observed by several authors (Wong and Hung, 2001; Blasco et al., 2012; Smoleń and Sady, 2012; Smoleń et al., 2014a).

Medrano-Macías et al. (2016) suggested that IO_3_^−^ could act as a substrate for widespread enzymes, such as nitrate reductase. Accordingly, Smoleń et al. (2011) reported that nitrate reductase could catalyse the reduction of IO_3_^−^ to I^−^, thus interfering with NO_3_^−^ metabolism in plants.

### Iodine Accumulation and Tolerance

Plants can absorb iodine by the roots as I^−^ through ionic channels and chloride transporters that are energized by proton pumps (White and Broadley, 2009). The lower toxicity of IO_3_^−^ observed in our work and in many other species (e.g.: barley, Duborská et al., 2018; cabbage, Weng et al., 2008; lettuce, Blasco et al., 2008; Blasco et al., 2010; Voogt et al., 2010; rice, Mackowiak and Grossl, 1999; spinach, Zhu et al., 2003; Smoleń et al., 2012; tomato, Caffagni et al., 2011; Kiferle et al., 2013) could be related to the necessity for IO_3_^−^ to undergo electrochemical or enzymatic reduction to I^−^ prior to plant uptake (Zhu et al., 2003; Mackowiak et al., 2005; Kato et al., 2013).

After root absorption, iodine is transported to the shoot mainly through the xylematic flux, while its redistribution through the phloem is low (Mackowiak and Grossl, 1999; Smolen et al., 2014a). Voogt et al. (2014) concluded that i) iodine accumulation in the shoot is dependent on the mass flow of water caused by plant *transpiration* and ii) the differences in iodine distribution across plant species and seasons could be explained by the difference in the transpiration rate.

In our work, iodine uptake and translocation to the leaves of both basil cultivars were dependent on the iodine form and concentration in the nutrient solution (**Figure 3**). The addition of KI to the nutrient solution produced an accumulation of iodine in the leaf tissue approximately eight-times higher than that induced by KIO_3_. At the same KI or KIO_3_ concentration in the nutrient solution, iodine tissue content in “Red Rubin” was similar or significantly higher than in “Tigullio” (**Table 2** and **Figures 3** and **4D**). This is an evidence that iodine tolerance in “Red Rubin” could not be ascribed to a root iodine-excluding mechanism or to a slower xylem loading of iodine than in “Tigullio,” and suggests that the higher iodine tolerance of “Red Rubin” was probably due to a superior ability to tolerate higher concentrations of iodine in leaf tissue.

Zhu et al. (2003) in hydroponically-grown spinach treated with 100 µM KI or KIO_3_, calculated the solution-to-leaf-transfer factor (TF_LEAF_) as the ratio between the iodine concentrations in leaves expressed on a fresh weight basis and in the nutrient solution: it was 19.4 and 2.2 for KI and KIO_3_, respectively. In our work (*Experiment 3*) TF_LEAF_ was 14.3 for 200 µM KI and 2.1 for 200 µM KIO_3_, in good agreement with the values reported by Zhu et al. (2003) for spinach.

Weng et al. (2008) demonstrated by electron microscopy that in cabbage roots iodine accumulated mainly in the cell wall, while in the leaves iodine was stored in the chloroplast. Some bibliographic evidences confirmed that iodide could easily be oxidized to elemental iodine, which can disrupt the cell membranes of roots and oxidize chlorophylls and carotenoids, thus resulting in leaf chlorosis and reduced CO_2_ assimilation (Lawson et al., 2015; Medrano-Macías et al., 2016; Duborská et al., 2018). Blasco et al. (2008; 2010) found that iodine application led to an increase in the concentrations of antioxidant molecules (e.g. ascorbic acid, glutathione, and phenolic compounds) and in the activity of some enzymatic antioxidants (e.g. superoxide dismutase, catalase, L-galactono dehydrogenase enzymes). In our work, we measured the concentration of total phenols and the antioxidant capacity in mature leaves, which were both higher in “Red Rubin” than in “Tigullio” and increased with leaf iodine content in both genotypes (**Figures 4D** and **6C, D**).

Phenols and anthocyanins are secondary metabolites involved in the plant protection against different types of biotic and abiotic stress, including mineral toxicity (Pardossi et al., 2015; Medrano-Macías et al., 2016). High leaf phenolic concentrations were thought to play an important role in boron tolerance of “Red Rubin” plants (Landi et al., 2014; Pardossi et al., 2015), and phenolic compounds can bind iodine through electrophilic substitution of H in the aromatic ring (Reiller et al., 2006). The anthocyanins contained in the epidermis of “Red Rubin” could act as a photoprotective layer for leaf mesophyll that partly absorbs the incident light energy and may diminish photooxidative damages of the chloroplasts (Landi et al., 2014; Pardossi et al., 2015). Based on these evidences, we can hypothesize that the same mechanisms could be involved also in the iodine tolerance of “Red Rubin.” Further work is necessary to verify this assumption.

### Iodine Biofiortification

In *Experiment 1*, we found that 10 µM KI did not affect plant growth and increased the leaf iodine content to 295 and 420 mg g^−1^ DW, respectively, in “Tigullio” and “Red Rubin” (**Table 2**). Considering a leaf dry matter content of 7.5% for basil leaves (on average), we calculated that the daily iodine RDA for healthy adults (150 µg day^−1^) could be satisfied by the assumption of approximately 6.7 g of fresh biofortified leaves of “Tigullio,” which corresponds to 7–8 leaves, an amount easy to add to a portion of salad.

Green-leaved sweet basil is also widely used to prepare the worldwide popular sauce “Genovese pesto.” Using the above-mentioned data, we calculated the hazardous amount, which is the maximum daily amount of sweet basil below the recommended dietary tolerable upper intake of iodine (1,100 µg day^−1^ for an adult of 70 kg body weight; Smoleń et al., 2019). For “Tigullio” containing 295 mg I kg^−1^ DW, the hazardous amount is 49.7 g of fresh leaves, which is approximately three-fold higher than the amount necessary to prepare a portion of pasta (80 g) dressed with “Genovese pesto” (about 12 g of sauce containing 6 g of fresh basil leaves).

## Conclusions

The detrimental effects of high iodine levels in the nutrient solution on the growth of both basil cultivars “Tigullio” and “Red Rubin” cultivated in hydroponic culture were associated to large iodine accumulation in leaf tissues and a marked reduction of leaf expansion and photosynthesis.

The greater tolerance to iodine toxicity of purple-leaved “Red Rubin” was associated with the ability to withstand higher concentrations of iodine in leaf tissues, rather than to an exclusion mechanism. High leaf concentration of phenolic compounds could play an important role in iodine tolerance of “Red Rubin.”

The supply of a nutrient solution containing 10 µM KI could be a simple and effective biofortification protocol for hydroponically-grown sweet basil. Our results also confirmed that this KI concentration is the maximum (in terms of consumer needs) recommended concentration that can be used to enrich sweet basil in iodine. Higher doses may already be harmful to the consumer.

## Author Contributions

LI: Substantial contributions to the conception or design of the work; drafting the work; interpretation of data and article writing; final approval of the version to be published; agreement to be accountable for all aspects of the work in ensuring that questions related to the accuracy or integrity of any part of the work are appropriately investigated and resolved. GC and RM: Performed the analyses of mineral ions, chlorophylls, carotenoids and growth analysis; support to article writing; revised the article critically; final approval of the version to be published. CP and CT: Performed the analyses of mineral ions, chlorophylls, carotenoids content in the plant and growth analysis. DS: Performed the analyses of mineral ions and growth analysis; interpretation of data. CK: Performed the analyses of antioxidant capacity, total phenol content, and growth analysis; support to article writing; revised the article critically; final approval of the version to be published. PP: support to article writing; revised the article critically; final approval of the version to be published. AP: conception or design of the work; analysis and interpretation of data; support to article writing; revised the article critically; final approval of the version to be published.

## Funding

This work was funded by University of Pisa.

## Conflict of Interest

The authors declare that the research was conducted in the absence of any commercial or financial relationships that could be construed as a potential conflict of interest.
