# Supplementary material for: Iodine Accumulation and Tolerance in Sweet Basil (Ocimum basilicum L.) With Green or Purple Leaves Grown in Floating System Technique
Source: Front Plant Sci. 2019 Dec 18;10:1494. doi: 10.3389/fpls.2019.01494 (PMC6930681; doi:10.3389/fpls.2019.01494)
Supplement: Supplementary file 1 [file Table_1.docx]

**Table SM1.** Influence of the concentration of potassium iodine (KI; control= 0.1μM) or potassium iodate (KIO_3_) in the nutrient solution on plant height, leaf area, root, stem, leaves and total dry weight (DW) of two cultivars (‘Tigullio’ and ‘Red Rubin’) of sweet basil (*Ocimum basilicum* L.). Plants were grown hydroponically under greenhouse e conditions for 21 days from 3^rd^ to 24^th^ June 2015 (Experiment [1](#_bookmark3)).

| **Cultivar** | **Treatment**  **(μM)** | | | **Root DW**  **(g plant^-1^)** | **Stem DW**  **(g plant^-1^)** | | **Leaf DW**  **(g plant^-1^)** | | **Total DW**  **(g plant^-1^)** | | | | **Plant height (cm plant^-1^)** | | **Leaf area**  **(cm^2^ plant^-1^)** | |  |
| --- | --- | --- | --- | --- | --- | --- | --- | --- | --- | --- | --- | --- | --- | --- | --- | --- | --- |
| ‘Tigullio’ | Control | | 0.77±0.15 a | | 4.24±0.43 a | | 6.18±0.56 a | | 11.17±0.82 a | | | | 61.1± 3.9 a | | 1605±192 a | | |
|  | 10 μM KI | | 0.67±0.09 ab | | 4.20±0.59 a | | 5.50±0.69 a | | 10.37±1.25 a | | | | 62.9±1.9 a | | 1584±220 a | | |
|  | 100 μM KI | | 0.51±0.16 b | | 0.85±0.15 d | | 2.07±0.52 b | | 3.43± 0.56 b | | | | 22.3±2.5 d | | 507±86 d | | |
|  | 10 μM KIO_3_ | | 0.68±0.11 ab | | 4.26±0.70 a | | 5.39±0.68 a | | 10.32±1.53 a | | | | 65.8±4.6 a | | 1672±152 a | | |
|  | 100 μM KIO_3_ | | 0.83±0.09 a | | 4.80±0.39 a | | 5.99±0.77 a | | 11.62±1.18 a | | | | 65.3±4.8 a | | 1536±170 a | | |
| ‘RedRubin’ | Control | | 0.18±0.04 c | | 1.25±0.27 b | | 1.87±0.25 bc | | 3.30±0.56 b | | | | 45.4± 8.5 b | | 880±105 b | | |
|  | 10 μM KI | | 0.16±0.03 c | | 1.13±0.23 bc | | 1.71±0.25 c | | 3.00±0.28 bc | | | | 43.4± 2.8 b | | 918±183 b | | |
|  | 100 μM KI | | 0.15±0.02 c | | 0.97±0.12 cd | | 1.43±0.29d | | 2.55±0.35 c | | | | 30.3± 2.5 c | | 764±83 c | | |
|  | 10 μM KIO_3_ | | 0.14±0.04 c | | 1.33±0.26 b | | 1.89±0.28 bc | | 3.36±0.42 b | | | | 44.8± 4.1 b | | 988±112 b | | |
|  | 100 μM KIO_3_ | | 0.14±0.05 c | | 1.15±0.22 bc | | 1.68±0.22 cd | | 2.97±0.46 bc | | | | 42.3±2.4 b | | 908±115 b | | |
| **Analysis of variance** | | | | | |  | |  | |  |  | | |  |  |  |  |
| Cultivar | |  | *** | | *** | | *** | | *** | | | *** | | | | *** |  |
| Treatment | |  | *** | | *** | | *** | | *** | | | *** | | | | *** |  |
| Cultivar×Treatment | | | *** | | *** | | ** | | *** | | | *** | | | | *** |  |

Significance: ** P<0.01; *** P<0.001. Values followed by the same letter within a column are not significantly different (*P*<0.05;Duncan’s test).

**Tab. SM2.** Effect of different concentrations of potassium iodide (KI) (Control=0.1 μM) or potassium iodate (KIO_3_) in the nutrient solution on root, stem, leaf, total biomass (dry weight, DW), plant height and leaf area of two cultivars (‘Tigullio’ and ‘Red Rubin’) of sweet basil (Ocimum basilicum L.). Each value is the mean (± SD) of three replicates. Plants were grown hydroponically under greenhouse conditions for 14 days from 9^th^ to 23^st^ May 2016 (Experiment 3).

| **Cultivar** | **Treatment**  **(μM)** | **Plant height (cm plant^-1^)** | **Root DW**  **(g plant^-1^)** | **Stem DW**  **(g plant^-1^)** | **Leaf DW**  **(g plant^-1^)** | **Total DW**  **(g plant^-1^)** | **Leaf area**  **(cm^2^ plant^-1^)** |
| --- | --- | --- | --- | --- | --- | --- | --- |
| ‘Tigullio’ | Control | 37.4±2.1 a | 0.64±0.09 a | 1.24±0.19 a | 2.32±0.33 a | 4.20±0.45 a | 966±103 a |
|  | 50 μM KI | 23.8±1.7 ef | 0.36±0.05 cd | 0.51±0.03 d | 1.51±0.10 cd | 2.38±0.14 c | 535±57 c |
|  | 100 μM KI | 15.6±1.1 g | 0.32±0.04 de | 0.33±0.05 f | 1.07±0.13 fg | 1.72±0.21 de | 304±43 d |
|  | 200 μM KI | 10.1±0.3 i | 0.26±0.04 ef | 0.18±0.10 h | 0.73±0.09 hi | 1.17±0.11 fg | 167±22 e |
|  | 100 μM KIO_3_ | 34.6±2.0 ab | 0.62±0.12 a | 1.08±0.18 a | 2.26±0.19 a | 3.96±0.43 ab | 930±85 a |
|  | 200 μM KIO_3_ | 31.3±1.5 b | 0.53±0.06 b | 0.87±0.07 b | 2.01±0.20 ab | 3.41±0.23 b | 701±56 b |
|  | 400 μM KIO_3_ | 22.4±1.6 f | 0.41±0.08 c | 0.57±0.11 d | 1.72±0.26 bc | 2.70±0.35 c | 512±58 c |
| ‘Red Rubin’ | Control | 32.1±1.0 b | 0.22±0.04 fg | 0.73±0.07 c | 1.42±0.17 de | 2.37±0.23 c | 663±74 b |
|  | 50 μM KI | 23.3±1.7 ef | 0.17±0.03 gh | 0.37±0.04 ef | 0.98±0.08 fgh | 1.52±0.14 def | 482±42 d |
|  | 100 μM KI | 16.8±1.0 g | 0.15±0.01 i | 0.35±0.03 h | 0.87±0.07 i | 0.83±0.10 g | 405±25 e |
|  | 200 μM KI | 12.9±0.7 h | 0.10±0.02 hi | 0.26±0.02 h | 0.73±0.07 i | 0.79±0.11 g | 377±18 e |
|  | 100 μM KIO_3_ | 27.1±2.3 d | 0.15±0.03 ghi | 0.49±0.09 de | 1.20±0.20 ef | 1.84±0.32 cd | 554±62 bc |
|  | 200 μM KIO_3_ | 25.3±0.5 de | 0.14±0.04 ghi | 0.47±0.08 def | 1.08±0.10 fg | 1.69±0.17 de | 492±71 c |
|  | 400 μM KIO_3_ | 22.3±1.2 f | 0.15±0.03 ghi | 0.34±0.06 f | 0.91±0.11 gh | 1.40±0.29 ef | 376±46 d |
| **Analysis of variance** | |  |  |  |  |  |  |
| Cultivar |  | *** | *** | *** | *** | *** | *** |
| Treatment |  | *** | *** | *** | *** | *** | *** |
| Cultivar×Treatment |  | *** | *** | *** | *** | *** | *** |

Significance: ** P<0.01; *** P<0.001. Values followed by the same letter within a column are not significantly different (*P*<0.05;Duncan’s test).:.

**Figure SM1.** Maas and Hoffman relationships (Y*= 100- s(X-t), see eq.6 for more details) for relative plant height (A) and total plant dry weight (B) versus the concentration of potassium iodide in the nutrient solution (Control=0.1, 10, 50, 100 or 200 μM) of two cultivars (‘Tigullio’ and ‘Red Rubin’) of sweet basil (Ocimum basilicum L.). Each value is the mean (± standard deviation, SD) of four replicates. The plants were grown hydroponically under greenhouse conditions for 16 days from 29^th^ June to 14^th^ July 2015 (Experiment 2).
